# Long range Debye-Hückel correction for computation of grid-based electrostatic forces between biomacromolecules

**DOI:** 10.1186/2046-1682-7-4

**Published:** 2014-06-17

**Authors:** Paolo Mereghetti, Michael Martinez, Rebecca C Wade

**Affiliations:** 1 Molecular and Cellular Modeling Group, Heidelberg Institute for Theoretical Studies (HITS), Schloß-Wolfsbrunnenweg 35, 69118 Heidelberg, Germany; 2 Center for Nanotechnology Innovation@NEST, Italian Institute of Technology, Piazza San Silvestro 12, Pisa, Italy; 3 Center for Molecular Biology (ZMBH), University of Heidelberg, Im Neuenheimer Feld 282, 69120 Heidelberg, Germany

**Keywords:** Continuum solvent electrostatics, Ionic strength, Debye-Hückel, Poisson-Boltzmann equation, Brownian dynamics simulation, Protein diffusion, Discretization grid, Finite difference, Second virial coefficient, Small angle scattering intensity

## Abstract

**Background:**

Brownian dynamics (BD) simulations can be used to study very large molecular systems, such as models of the intracellular environment, using atomic-detail structures. Such simulations require strategies to contain the computational costs, especially for the computation of interaction forces and energies. A common approach is to compute interaction forces between macromolecules by precomputing their interaction potentials on three-dimensional discretized grids. For long-range interactions, such as electrostatics, grid-based methods are subject to finite size errors. We describe here the implementation of a Debye-Hückel correction to the grid-based electrostatic potential used in the SDA BD simulation software that was applied to simulate solutions of bovine serum albumin and of hen egg white lysozyme.

**Results:**

We found that the inclusion of the long-range electrostatic correction increased the accuracy of both the protein-protein interaction profiles and the protein diffusion coefficients at low ionic strength.

**Conclusions:**

An advantage of this method is the low additional computational cost required to treat long-range electrostatic interactions in large biomacromolecular systems. Moreover, the implementation described here for BD simulations of protein solutions can also be applied in implicit solvent molecular dynamics simulations that make use of gridded interaction potentials.

## Background

Simulations of concentrated solutions of macromolecules such as those designed to mimic the intracellular environment, are becoming feasible due to improvements in computational power and simulation methods [1-5]. Given that even for simulating a small volume of a protein solution, several hundreds of proteins have to be taken into account, coarse grained methods, which neglect atomic details, e.g. by treating each protein as a sphere, are often applied [6].

However, to understand the effects of differences in protein sequence or point mutations from simulations requires a more detailed level of modeling. Explicit inclusion of atomic detail can be computationally demanding and therefore, approximations and calculation strategies are required to make the simulations feasible. A commonly employed approach is to retain atomic detail for the macromolecules while treating them as rigid bodies in continuum solvent. Apart from restricting the number of degrees of freedom considered in the simulations, this treatment permits interaction forces between macromolecules to be computed efficiently by precomputation of their interaction potentials on three-dimensional discretized grids. Thus, during the simulations, forces can be computed by considering the interactions of each atom of each macromolecule with the interaction potential grids of the other macromolecules. Grid formalisms for intermolecular interactions are extensively used for macromolecular docking methodologies [7,8], binding site determination [9], as well as in structure determination from electron microscopy maps [10,11]. A major drawback of gridded potentials is, however, the occurrence of finite size problems [3]. To minimize truncation errors in computing energies or forces, the interaction potential must be small at the edges of a grid. For molecular electrostatic potentials, the long-range nature of the Coulombic interaction, especially at low salt concentration or for highly charged macromolecules, means that very large grids are often required. For example, at 5 mM ionic strength, the Debye length of the solution is 43 Å. For a small globular protein with a radius of 20 Å and a net charge of + 10*e*, the electrostatic grid dimensions should be at least 200 × 200 × 200 Å to obtain an electrostatic potential of ≈ 0.1 kcal/mol/*e* at the grid edges. Assuming a grid spacing of 1 Å, the grid must have at least 201 × 201 × 201 points. This grid size is not a problem when a single small protein is considered but becomes an issue when simulating a periodic box containing several hundreds or thousands of proteins in solution. The grid size can also be a problem for memory usage in calculations for one or a few large macromolecules.

One solution to this problem is to use multiple focused grids with different grid spacings centered on each macromolecule: a detailed potential grid with a small grid spacing for representing the electrostatic potential at short-range and a coarse grid with a larger grid spacing for the long-range part [1]. Another solution, which will be described in this paper, is to exploit the fact that beyond a certain distance from the surface of the macromolecule, the electrostatic potential becomes centrosymmetric. Thus, a cubic gridded potential is used for the short-range part of the electrostatic potential up to a defined distance threshold and a continuous screened Coulomb potential is used beyond this distance. The distance threshold corresponds to the radius of the largest sphere enclosed by the grid.

We have recently developed a Brownian dynamics (BD) method for simulating many macromolecules (10^2^-10^3^) described as atomically detailed rigid bodies in a continuum solvent in a periodic box [3]. The model used is based on that originally developed for the simulation of the diffusional association of two proteins and implemented in the SDA (Simulation of Diffusional Association) software [8]. For the simulation of many proteins, this method gives results in good agreement with experimental translational and rotational diffusion coefficients and small angle scattering structure factors for dilute [3] as well as concentrated protein solutions [12]. In this approach, intermolecular forces are computed as the sum of electrostatic interaction, electrostatic desolvation, non-polar desolvation and soft-core repulsion terms [3,8]. For computational efficiency, all these terms are precomputed on grids for each macromolecular solute before carrying out the BD simulations. To overcome errors due to the finite size of the electrostatic grids, we here describe the implementation of a long-range electrostatic correction to the model for interaction forces used in our BD simulations. The purpose of this correction is to improve the accuracy of the computed inter-protein forces and extend the applicability of the approach to highly charged proteins and low ionic strength conditions. For validation, we performed BD simulations of bovine serum albumin (BSA) and hen egg white lysozyme (HEWL) with and without the long-range electrostatic correction and compared the results to experimentally determined small angle scattering structure factors and self-diffusion coefficients. The same methodology described here for the implementation of the long-range Debye-Hückel correction, should also be applicable in implicit solvent molecular dynamics simulations that make use of gridded interaction potentials [13-16].

## Methods

Brownian dynamics (BD) is a simulation method that employs a mesoscopic model in which the solvent is treated as a continuum and the solutes are modelled as discrete entities at a level of detail appropriate for the problem being studied. BD thus takes advantage of the large separation in timescale between the fast solvent motion and the slower motion of solute particles (polymers or colloids) which make it possible to treat the solvent implicitly. Furthermore, internal solute degrees of freedom are often neglected and macromolecules are treated as rigid bodies interacting by direct interactions (electrostatic, van der Waals, non-polar) and solvent-mediated (hydrodynamic) interactions. Due to these simplifications, BD can be used to study larger biomacromolecular systems on longer time-scales than is possible with classical atomic-detail molecular dynamics simulations.

Translational motion is propagated according to the following Equation [17]:

(1) $$r_{i}(t_{1})=r_{i}(t_{0})+\underset{j}{\sum }\frac{\partial D_{\text{ij}}^{t}}{\partial r_{j}(t_{0})}\Delta t+\underset{j}{\sum }\frac{D_{\text{ij}}^{t}}{\text{kT}}F_{j}(t_{0})\Delta t+R_{i}$$

where *r*_*i *_is the position of the center of geometry of the solute *i* and *Δt *= (*t*_1 _- *t*_0_) is the timestep.

The effect of the solvent is described by a random displacement, **R**_*i*_, which mimics the collision of solute *i* with the solvent molecules and is defined by a Gaussian distribution with mean 〈**R**_*i*_〉= 0 and covariance $\langle R_{i}R_{j}\rangle =2D_{\text{ij}}^{t}\mathrm{\Delta t}$. From the latter, it follows that the stochastic displacement is proportional to the square root of the translational diffusion tensor, $D_{\text{ij}}^{t}$. The second term on the r.h.s. of Equation 1, the divergence of the diffusion tensor, describes the hydrodynamic drift of the solute towards regions of high mobility. The force acting on solute *i* results from the sum of the forces acting on solutes *j* at time *t*_0_, **F**_*j*_(*t*_0_), coupled with the diffusion tensor.

We employ a simplified treatment of hydrodynamic interactions to avoid the computationally expensive Cholesky factorization required to calculate the square root of the diffusion matrix. A mean field approach is used where $D_{\text{ij}}^{t}$ is replaced by a volume fraction-dependent diffusion coefficient, $D^{t_{\text{short}}}(\phi_{i})$, and Equation 1 simplifies to [12]

(2) $$r_{i}(t_{1})=r_{i}(t_{0})+\frac{D^{t_{\text{short}}}(\phi_{i})}{\text{kT}}F_{i}(t_{0})\Delta t+R_{i}$$

We define the local volume, *V*_*i*_, as the volume of the sphere of radius *R*^*cut *^centered on solute *i*. The local volume fraction *ϕ*_*i *_for the solute *i* is obtained by dividing the sum of the volumes of the solutes within *R*^*cut *^by the local volume *V*_*i*_[18]. The volume of a protein, *v*, is computed by approximating the protein as a sphere having a radius equal to the hydrodynamic radius (*σ*^*stokes*^) estimated using HYDROPRO [19]. The cutoff for the local volume, *R*^*cut*^, is set to four times the side of the largest interaction grid of the central solute. For a small simulation box, this cutoff was rescaled to a value equal to half of the simulation box size. A solute *j* is totally included in the local volume when the center-to-center distance *d*_*ij *_between the central solute *i* and solute *j* is less than $R_{\text{cut}}-\sigma_{j}^{\text{stokes}}$. When a solute *k* is only partially included within *R*^*cut*^, that is, when $R^{\text{cut}}-\sigma_{k}^{\text{stokes}}<d_{\text{ik}}<R^{\text{cut}}+\sigma_{k}^{\text{stokes}}$, we account for that portion of solute volume derived by the sphere-sphere intersection. The volume fraction dependent short-time translational diffusion coefficient ($D^{t_{\text{short}}}(\phi_{i})$) is then obtained using the Tokuyama model [20-22], derived for a concentrated hard-sphere suspension of particles interacting with both direct and hydrodynamic interactions. An equation analogous to Equation 2 is used for the rotational motion [12], with the volume fraction dependent short-time rotational diffusion coefficient obtained using the model derived by Cichocki et al. which includes lubrication forces as well as two- and three-body expansions of the mobility functions [23].

The forces, *F*_*i*_, are computed as finite-difference derivatives of the pairwise free energies of interaction between the solutes as described in the next section.

### Interaction energies and forces

For each pair of macromolecules, the interaction free energy, *ΔG*^1-2^, is defined as:

(3) $$\begin{array}{lll} \Delta G^{1-2}= & \;\frac{1}{2}\underset{i_{2}}{\sum }\Phi_{el_{1}}(r_{i_{2}})\cdot q_{i_{2}}\\ & +\frac{1}{2}\underset{j_{1}}{\sum }\Phi_{el_{2}}(r_{j_{1}})\cdot q_{j_{1}} & \text{[electrostatic interaction]}\\ & +\underset{i_{2}}{\sum }\Phi_{\text{edesol}v_{1}}(r_{i_{2}})\cdot q_{i_{2}}^{2}\\ & +\underset{j_{1}}{\sum }\Phi_{\text{edesol}v_{2}}(r_{j_{1}})\cdot q_{j_{1}}^{2} & \text{[electrostatic desolvation]}\\ & +\underset{m_{2}}{\sum }\Phi_{\text{npdesol}v_{1}}(r_{m_{2}})\cdot \text{SAS}A_{m_{2}}\\ & +\underset{n_{1}}{\sum }\Phi_{\text{npdesol}v_{2}}(r_{n_{1}})\cdot \text{SAS}A_{n_{1}} & \text{[non-polar desolvation]}\\ & +\underset{m_{2}}{\sum }E_{\text{softcor}e_{1}}(r_{m_{2}})\\ & +\underset{n_{1}}{\sum }E_{\text{softcor}e_{2}}(r_{n_{1}}) & \text{[soft-core repulsion]} \end{array}$$

A detailed description and parameterization of Equation 3 can be found in Refs. [3,24]. Briefly, the first two terms in Equation 3 are the interaction energies of the charges of one macromolecule ($q_{i_{2}}$ or $q_{j_{1}}$) with the electrostatic potential of the other macromolecule ($\Phi_{el_{1}}$ or $\Phi_{el_{2}}$). Charges were assigned using the effective charge approximation [25]. The third and fourth terms of Equation 3 represent the electrostatic desolvation energy arising from the introduction of the low dielectric cavity of one macromolecule in the presence of the charges of the other [25,26]. The desolvation energy is computed as the interaction of the charges of one macromolecule ($q_{i_{2}}$ or $q_{j_{1}}$) with the electrostatic desolvation potential of the other macromolecule ($\Phi_{\text{edesol}v_{1}}$ or $\Phi_{\text{edesol}v_{2}}$) [26], with parameterization as in Ref. [24]. The fifth and sixth terms in Equation 3 correspond to the non-polar interactions due to the burial of the solvent accessible surface areas (SASAs) of the surface atoms The last two terms of Equation 3 describe the soft-core repulsive potential introduced to avoid overlaps. The soft-core potential is modelled using an inverse power function. The smoothness of the soft-core potential allows abrupt changes in the forces at close contact to be avoided. In Equation 3, **r** specifies the atomic coordinates. For computational efficiency, all interaction potentials, *Φ*, are mapped onto grids centered on each of the macromolecules.

This formalism implies a truncation of the electrostatic potential in the grid-charge formalism due to the finite extent of the grids. To alleviate this problem, we here introduce an analytical long-range correction to the electrostatic interaction term that makes use of the assumption that beyond the electrostatic grid boundaries, a macromolecule can be treated as a Debye-Hückel sphere.

According to the Debye-Hückel theory of dilute electrolyte solutions, all ions in the solvent are treated as point charges while each pair of solutes are treated as spheres with radii *a*_*i*_, *a*_*j *_and net charges *z*_*i*_*e*_*l*_, *z*_*j*_*e*_*l*_, where *e*_*l *_is the elementary charge. Then, the potential of mean force between a pair of solute molecules is

(4) $$\begin{array}{l} \begin{array}{lll} w(r) & =+\infty & r<a\\ =\frac{z_{i}z_{j}e_{l}^{2}e^{-\kappa (r-a)}}{4\pi \varepsilon_{0}\varepsilon_{r}r(1+\mathrm{\kappa a})} & r\geq a \end{array} \end{array}$$

where *ε*_0_ is the vacuum permittivity, *ε*_*r *_is the relative permittivity of the solvent, *a *= *a*_*i *_+ *a*_*j*_, and *κ* is the inverse of the Debye length, and is proportional to the ionic strength $\left(\kappa^{2}=\frac{e_{l}^{2}\beta }{\varepsilon_{0}\varepsilon_{r}}\underset{i}{\sum }\rho_{i}z_{i}^{2}\right)$.

As shown in Equation 3, to compute the electrostatic interaction between a pair of macromolecules, the electrostatic potential of macromolecule 1 is multiplied by the effective charges of the second macromolecule. Due to the finite size of the grid, when the second macromolecule is on the border of the electrostatic potential grid of macromolecule 1, only a fraction of the effective charges on macromolecule 2 are taken into account for computing the electrostatic interaction. An isotropic distance cut-off from the center of macromolecule 1 is used in computing this interaction, so that if the effective charge is beyond this distance cutoff, its electrostatic interaction is not computed. The spherical cut-off is assigned on the assumption that the electrostatic potential becomes centrosymmetric at the grid edges and therefore a switch to the analytical Debye-Hückel potential can be made beyond the cutoff. The application of the Debye-Hückel potential reduces the discontinuity in the energy and forces at the grid cut-off distance.

### Second osmotic virial coefficients

Osmotic virial coefficients are coefficients in the virial expansion of the state equation and they reflect deviations from ideal behaviour due to the presence of interactions. For simple cases, they can be obtained analytically. For this reason, they are commonly used to assess force field accuracy [1,3,27,28].

From classical statistical mechanics, the second osmotic virial coefficient can be obtained from [29]

(5) $$B_{22}=-\frac{1}{2V}\overset{\infty }{\underset{0}{\int }}\left[e^{-\frac{w(r)}{k_{B}T}}-1\right]d\Omega$$

Where *r* is the center-to-center distance and *w *(*r*) is the potential of mean force. For an isotropic potential, the corresponding equation is

(6) $$B_{22}=-\frac{1}{2}\overset{\infty }{\underset{0}{\int }}\left[e^{-\frac{w(r)}{k_{B}T}}-1\right]4\pi r^{2}\text{dr}$$

### Small angle scattering intensity

To assess the correctness of the interaction potentials, we compared experimental and computed small angle scattering intensities. Scattering intensities were computed from the simulations using [30]

(7) $$I(q)=\gamma n_{p}{\left(\Delta \Delta \right)}^{2}v^{2}P(q)S(q)$$

where *γ* is a factor related to instrument effects, *n*_*p *_= *N*/*V* is the protein concentration expressed as number density (*N* is the number of particles and *V* the total volume of the solution), *Δ ρ * is the electron density contrast between the scattering particle and the solvent, and *v* is the particle volume. *P *(*q*) is the form factor normalized such that *P*(0) = 1, *S *(*q*) is the structure factor and *q* is the scattering vector. The pre-factor *γ *(*Δρ*)^2^*v*^2^ can be obtained in experiments and then the normalized scattering intensity is expressed as

(8) $$\frac{I(q)}{An_{p}}=P(q)S(q),\;\text{where}A=\gamma {\left(\Delta \Delta \right)}^{2}v^{2}$$

We computed the form factor for BSA using the analytical expression for the orientationally averaged form factor of an oblate ellipsoid with radii *a* and *b* where *a* is the semi-axis of revolution [31,32]. Following ref. [32], we set *a *= 17.5 Å and *b *= 47.4 Å.

The structure factor, *S *(*q*), was computed by Fourier transformation of the radial distribution function, *g *(*r*) [33] as follows

(9) $$S(q)=1+4\pi n_{p}\overset{\infty }{\underset{0}{\int }}h(r)\frac{\text{sin}(\text{rq})}{\text{rq}}r^{2}\text{dr}$$

where *n*_*p*_ is the number density, *r* is the center-to-center distance, *q* is the magnitude of the scattering vector given by *q*=4*πλ*^-1^*sin *(*θ*/2) (where *θ* is the total scattering angle) and *h *(*r*) is the total correlation function which is given by *h *(*r*) = *g*(*r*) - 1. The radial distribution function was computed from BD simulations using the center-to-center protein distances. We estimated the convergence of the *g *(*r*) by checking that it was not varying with increasing simulation time. This was done by computing the *g *(*r*) over the full trajectory and comparing this *g *(*r*) with an average *g *(*r*) computed from 20 segments selected sequentially from the trajectory.

### Test systems of two spherical particles

For a system composed of two charged soft-sphere particles interacting via a Debye-Hückel potential, the long-range contribution to the second virial coefficient can be computed by integrating Equation 6. This equation can be solved analytically by expanding the exponential $e^{-w(r)/k_{B}T}$ up to the second order and substituting the Debye-Hückel expression for the potential of mean force [29,34].

Only the long-range contribution to the second virial coefficient is taken into account in the analysis. Hence, the lower bound of the integration (*lb*) is not 0 but it is set to the sum of the protein radii (*a*_*i *_+ *a*_*j*_) plus one or two Debye lengths (1/*κ*). For example, solving Equation 5 setting the lower bound to *lb *= (*a*_*i *_+ *a*_*j*_) + 1/*κ* gives

(10) $$\;B_{22}^{1/\kappa }=\frac{z_{i}z_{j}e_{l}^{2}}{2e\rho }\left[\frac{2+\kappa a}{1+\kappa a}-\frac{\kappa }{4\pi \varepsilon_{0}\varepsilon_{r}\text{ekT}}\frac{z_{i}z_{j}e_{l}^{2}}{{\left(1+\kappa a\right)}^{2}}\right]$$

where *e* is the base of the natural logarithm, *e*_*l *_is the elementary charge and *ρ* is the concentration of the ions (equivalent to the ionic strength for monovalent ions).

The reason for considering only the long-range contribution is two-fold. Firstly, our purpose is to assess the accuracy of the long-range Debye-Hückel potential included in the BD simulation model. Secondly, for the expansion of the exponential *e*^-*w*/*k* *T*^ up to the second order to be reasonably accurate, |*w*/*kT*| ≪ 1 is required. This means that the short-range contribution of *B*_22_ at low ionic strength or for highly charged systems cannot be obtained using Equation 5.

In the numerical integration, the two particles were represented by spherical fullerene-like particles of radius 6 Å composed of 180 atoms. A partial point charge was placed on each atom. The total charge of each sphere was uniformly distributed over all the atoms. Different systems were simulated by varying the net charge and the ionic strength (see Table 1 and Table 2 in Results and discussion). The interaction energy between the two particles is given by

**Table 1:** **Long range contribution to the** ***B***_**22**_** value at 5 mM ionic strength for the two soft-sphere systems**

***z***_***i***_	***z***_***j***_		$B_{22}^{\left(EP_{100A}\right)}$		$B_{22}^{\left(EP_{200A}\right)}$		$B_{22}^{\left(EP_{100A}+\text{DHP}\right)}$		$B_{22}^{\left(A\right)}$
**(*e***_***l***_**)**	**(*e***_***l***_**)**	**1/*κ***	**2/*κ***	**1/*κ***	**2/*κ***	**1/*κ***	**2/*κ***	**1/*κ***	**2/*κ***
+1	+1	0.0	0.0	13.0^(0.0)^	0.0	29.0^(0.0)^	14.1^(0.0)^	30.4	16.1
+1	-1	0.0	0.0	-13.3^(0.0)^	0.0	-29.4^(0.0)^	-14.2^(0.0)^	-30.7	-16.2
+5	+5	0.0	0.0	261.5^(0.1)^	0.0	636.9^(0.1)^	339.3^(0.2)^	675.8	397.4
+5	-5	0.0	0.0	-432.7^(0.3)^	0.0	-853.8^(0.3)^	-367.8^(0.3)^	-878.0	-429.8
+10	+10	0.0	0.0	603.6^(0.2)^	0.0	1916.9^(0.2)^	1216.5^(0.7)^	1490.0	1428.1
+10	-10	0.0	0.0	-5037.6^(9.7)^	0.0	-7338.9^(10.5)^	-1682.2^(1.7)^	-4738.0	-1867.6

Computed values with electrostatic potential grids only (EP) are compared to the computed values with electrostatic potential grids plus the Debye-Hückel potential (EP+DHP) and to the analytical values (A). Lower bounds to integrate the *B*_22_ were assigned as one or two Debye lengths (1/ *κ*), see manuscript for details. The *B*_22_ values are given in units of (mol mL g ^-2^) x 10 ^4^ with standard deviations in parentheses.

**Table 2:** **Long range contribution to the *B***_**22 **_**values at 300 mM ionic strength for the two soft-sphere systems**

***z***_***i***_	***z***_***j***_		$B_{22}^{\left(EP_{100A}\right)}$		$B_{22}^{\left(EP_{200A}\right)}$		$B_{22}^{\left(EP_{100A}+\text{DHP}\right)}$		$B_{22}^{\left(A\right)}$
**(*e***_***l***_**)**	***(e***_***l***_ ***)***	**1/*κ***	**2/*κ***	**1/*κ***	**2/*κ***	**1/*κ***	**2/*κ***	**1/*κ***	**2/*κ***
+1	+1	0.3^(0.0)^	0.1^(0.0)^	0.3^(0.0)^	0.1^(0.0)^	0.3^(0.0)^	0.1^(0.0)^	0.3	0.1
+1	-1	-0.3^(0.0)^	-0.1^(0.0)^	-0.3^(0.0)^	0.1^(0.0)^	0.3^(0.0)^	0.1^(0.0)^	-0.3	-0.1
+5	+5	6.4^(0.0)^	3.3^(0.0)^	6.6^(0.1)^	3.5^(0.0)^	6.7^(0.0)^	3.6^(0.0)^	7.4	4.0
+5	-5	-9.4^(0.0)^	-3.7^(0.0)^	-9.6^(0.2)^	-3.9^(0.1)^	-9.7^(0.1)^	4.0^(0.1)^	-11.6	-4.6
+10	+10	18.1^(0.0)^	11.4^(0.0)^	18.8^(0.1)^	12.0^(0.1)^	19.4^(0.1)^	12.6^(0.1)^	4.2	12.8
+10	-10	-97.6^(0.7)^	-18.4^(0.1)^	-98.1^(0.4)^	-19.0^(0.1)^	-98.9^(0.7)^	-19.6^(0.1)^	-72.4	-22.0

Computed values with electrostatic potential grids only (EP) are compared to the computed values with electrostatic potential grids plus the Debye-Hückel potential (EP+DHP) and to the analytical values (A). Lower bounds to integrate the *B*_22_ were set to one or two Debye lengths (1/ *κ*), see manuscript for details. *B*_22_ values are given in unit of (mol mL g ^-2^) x 10 ^4^ with standard deviations in parentheses.

(11) $$\begin{array}{lll} \Delta G_{\text{Debye}}^{1-2}= & \;\frac{1}{2}\underset{i_{2}}{\sum }\Phi_{el_{1}}(r_{i_{2}})\cdot q_{i_{2}}\\ & +\frac{1}{2}\underset{j_{1}}{\sum }\Phi_{el_{2}}(r_{j_{1}})\cdot q_{j_{1}} & \text{[electrostatic]}\\ & +\frac{z_{i}z_{j}e_{l}^{2}e^{-\kappa (r-a)}}{4\pi \varepsilon_{0}\varepsilon_{r}r(1+\kappa a)} & \text{[Debye-Hückel ]}\\ & +\underset{m_{2}}{\sum }E_{\text{softcor}e_{1}}(r_{m_{2}})\\ & +\underset{n_{1}}{\sum }E_{\text{softcor}e_{2}}(r_{n_{1}}) & \text{[soft-core repulsion]} \end{array}$$

To compute the second virial coefficient, one particle was kept fixed at the center of the simulation box and the other was moved on a regular lattice within the simulation box, avoiding overlaps with the central particle. The size of the box was set to 400×400×400 Å ^3^ and the dimension of the lattice was set to 100×100×100 vertices. The interaction energy (Equation 11) was computed for each position assumed by the second particle and the second virial coefficient was computed by integrating Equation 6 numerically with the potential of mean force, $w(r)=\Delta G_{\text{Debye}}^{1-2}$, where *r* is the center-to-center distance. As for the analytical computation of *B*_22_, the integration was performed setting half, one, or two Debye lengths as the lower bound of the integral.

We considered two spherical particles *i* and *j* with corresponding radii *a*_*i*_ and *a*_*j*_ and net charges *z*_*i*_ and *z*_*j*_, each resulting from 180 partial point charges uniformly distributed near the surface of each particle at a distance *r* from the particle’s center. Six different combinations of net charges on the particles were tested, namely: + 1/ + 1, + 5/ + 5, + 10/ + 10 and + 1/ -1, + 5/ -5, + 10/ -10 (in units of elementary charge). For each pair of particles the integration was performed at different ionic strengths, 5 mM and 300 mM. These two ionic strengths were chosen to assess the importance of the Debye-Hückel term at low and high salt conditions (compared to the 150 mM physiological ionic strength). The computed values were obtained by with and without inclusion of the Debye-Hückel potential.

From the set of approximately 10^6^ interaction energies computed at the lattice vertices (avoiding the overlapping region), we extracted 100 random subsets of 10^5^ values. For each subset, the second virial coefficient was computed. Then, an average *B*_22_ and a standard deviation over the subset was calculated.

### BD Simulations of protein solutions

BD simulations were performed with SDAMM [3], a parallelized program based on the SDA software [8] capable of handling many proteins (10^3^- 10^4^) treated as rigid bodies in atomic detail. For further details, see [3].

BD simulations were carried out for 250 protein molecules that were initially randomly positioned (avoiding overlaps) in a cubic box with periodic boundary conditions. The dimensions of the simulation box were varied according to the concentration of the protein solution.

The Debye-Hückel interaction between a pair of proteins was computed up to a distance cutoff of 4 times the side of the electrostatic grid. If the simulation box was small, to avoid self-image interactions, this cutoff was rescaled to a value equal to half of the simulation box size.

Each system was subjected to 5 or 10 *μ*s of simulation at 300 K. Equilibration was assessed by monitoring the convergence of the radial distribution function and the stabilization of the energies. In all cases, 1 *μ*s was sufficient to obtain an equilibrated system according to these criteria and the remaining 4 or 9 *μ*s were used for the analysis. The integration timestep was 0.5 ps. The positions and orientations of the proteins were recorded along with energy values every 0.5 ns.

Simulations of HEWL were performed at 14, 28, 57 and 85 g/L for comparison with experimental long-time translational self-diffusion coefficients [35]. Four sets of simulations were performed varying the ionic strength (1 mM and 5 mM) and including or omitting the analytical Debye-Hückel potential. Simulations were performed for 5 *μs*.

Simulations of BSA were performed at 0.9, 4.5, 9, 18, 45, 90 g/L for comparison with the experimental small angle X-ray scattering (SAXS) intensities described in ref. [32]. Two sets of simulations were performed. In one set, the Debye-Hückel potential was included, whereas in the other set, the Debye-Hückel potential was omitted. Because of the faster convergence of the higher concentration simulations, simulations at 0.9, 4.5, 9 and 18 g/L were performed for 10 *μs* whereas the simulations at 45 and 90 g/L were performed for 5 *μs*.

### Protein preparation

The crystal structure of hen egg white lysozyme (HEWL) was taken from the Protein Data Bank (ref): 1hel. The structure of BSA used for the simulations was a model taken from Modbase [36]. It was obtained by homology modelling based on the crystal structure of human serum albumin (HSA) [37].

Polar hydrogen atoms were added to the structures according to the specified pH and ionic strength (IS) using the H++ software [38]. The simulations of HEWL were performed at pH 5 ; the computed net charge of HEWL was +10*e*. The simulations of BSA were performed at pH 7. BSA had a computed net charge of -16*e*.

Atomic partial charges and radii were assigned to all the atoms from the OPLS united atom force field [39]. Electrostatic potential grids *Φ* were computed by solving the linearized Poisson-Boltzmann equation using the program UHBD [40]. The grid size was set to 100×100×100 Å ^3^ for HEWL and 200×200×200 Å ^3^ for BSA with a grid spacing of 1.0 Å. Non-polar desolvation, electrostatic desolvation and soft-core repulsion grids were set to 100×100×100 Å ^3^ for HEWL and 130×130×130 Å ^3^ for BSA, with a grid spacing of 1.0 Å.

## Results and discussion

### Comparison of simulations and analytical results for systems of two spherical particles

The two spheres system (see Computational Details section) was simulated with different combinations of net solute charge at two ionic strengths with and without inclusion of the Debye-Hückel potential. For each system, the analytical value of the long range contribution to the *B*_22_ was compared to the computed one. All values are given in Table 1 for 5 mM and Table 2 for 300 mM ionic strength. For a better comprehension of the length scale of the contribution of the electrostatic potential to the second virial coefficient, the analytical *B*_22_ values from the analytical calculations and from the simulations were obtained using different lower bounds for integrating Equation 6. We first consider the systems at low ionic strength (5 mM).

#### ***5 mM ionic strength***

Let us first consider the integration done with a lower bound of one Debye length which at 5 mM ionic strength corresponds to 43 Å. From Table 1, it is clear that when using a grid of 100×100×100 Å ^3^ without the Debye-Hückel potential, the long-range decay of the electrostatic potential is not captured. This result is expected since the electrostatic potential grid size is of the same order as the Debye length. Doubling the length of the side of the grid results in a *B*_22_ value that is approximately 50% of the analytical value. The long-range tail (beyond 100 Å) of the electrostatic potential is missing and it is apparent that it represents an important contribution to the second virial coefficient.

By turning on the Debye-Hückel potential and keeping the smaller electrostatic potential grid (side length: 100 Å), more than the 90% of the analytical *B*_22_ value is recovered. For systems with the highest net charge at one Debye length, the potential is too high and the integral expression in Equation 6 diverges.

For a perfectly isotropic case, such as this one, the Debye-Hückel potential smoothly recovers the truncation of the electrostatic potential due to the finite grid. This can be seen from the electrostatic potential energy computed by varying the inter-particle separation (see Additional file 1).

At two Debye lengths (2/*κ*), the *B*_22_ value of the systems with the smaller grid (100 Å) without the Debye-Hückel potential is zero, since the grid is smaller than the Debye length. By doubling the grid dimension, the side of the grid becomes of the same order as the Debye length and the *B*_22_ is still not computed correctly. With the Debye-Hückel potential and the smaller grid, however, the analytical second virial coefficient can be reproduced well.

#### ***300 mM ionic strength***

Increasing the ionic strength to 300 mM, at lower bounds of one or two Debye lengths (5.5 Å), the *B*_22_ values computed using only the smaller electrostatic potential grid agree rather well with the analytical values, see Table 2. Doubling the grid dimensions or adding the Debye-Hückel potential is not required because more than 90% of the interactions are captured within one Debye length. It is clear that at 300 mM ionic strength, the grid-based formalism is sufficient to properly describe the long-range electrostatic interaction, even using the smaller grid.

### Protein systems modeled in atomic detail

We now turn to more complex and realistic systems composed of solutions of proteins represented in atomic detail subjected to BD simulation as described in the Computational Details section.

#### 

##### 

**Scattering intensities** Several BSA solutions at different concentrations were simulated for 10 *μs* to 20 *μs* using BD. To assess the effect of the Debye-Hückel approximation on the BSA self-interactions, two sets of simulations were performed. In one set, the Debye-Hückel potential was included whereas in the other set, it was omitted.

Normalized small angle scattering intensities were computed using Equation 8 and compared to experimental SAXS intensities. The experiments were performed without added salt which corresponds to an ionic strength up to 5 mM [31,32]. This non-zero ionic strength arises from several factors such as dissolved CO _2_, a residual amount of salt present in the protein solution, and the dissociation of surface groups upon solvation [31,32]. Simulations were performed at 5 mM ionic strength with a corresponding Debye length of 43.1 Å.

**Figure 1:**
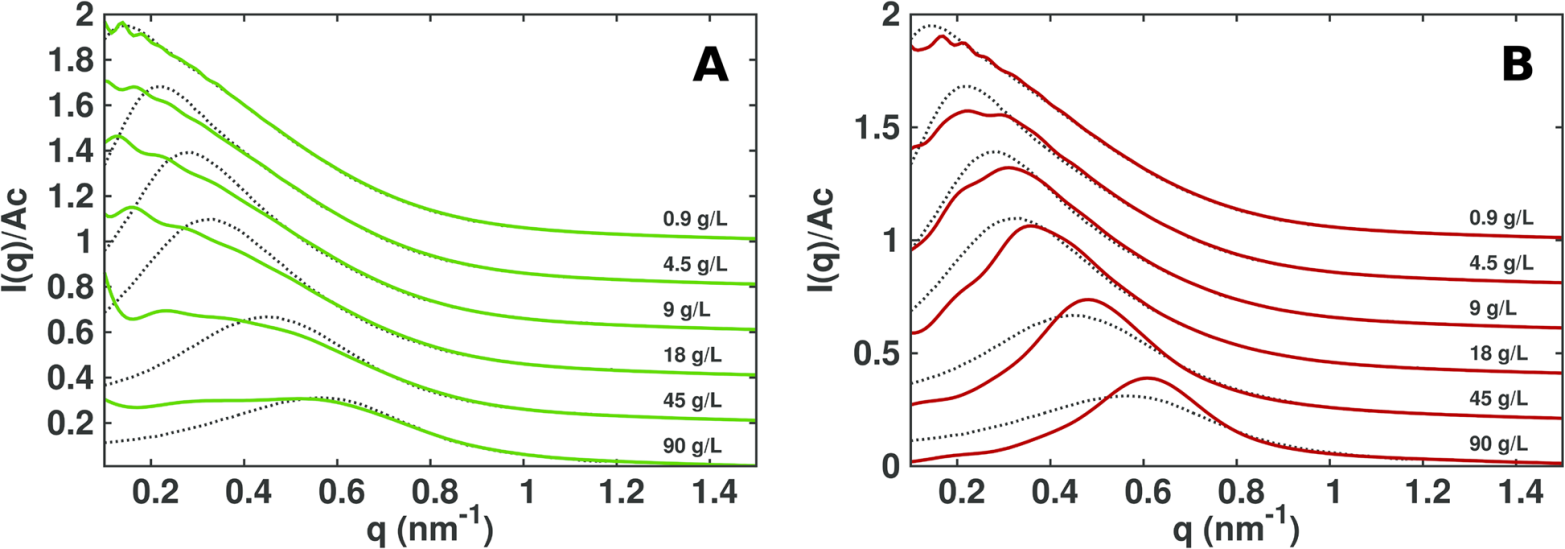
**BSA SAS intensities.** Experimental [32] (dashed lines) and computed (continuous lines) normalized small angle scattering intensities at different concentrations (indicated on the plots) of BSA. Computed curves from simulations without **(A)** and with **(B)** the Debye-Hückel approximation. Curves are shifted by 0.2 on the vertical axis for better visibility.

**Figure 2:**
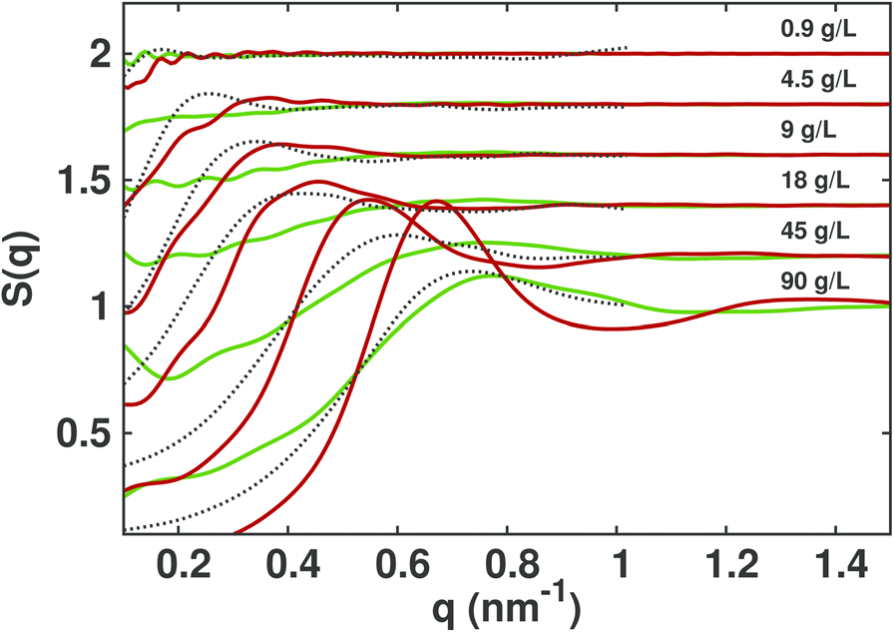
**BSA structure factors.** Experimental [32] (dashed lines) and computed (continuous lines) structure factors at various concentrations (indicated on the plot) of BSA obtained from simulations without (dark green) and with (dark red) the Debye-Hückel approximation. Curves are shifted by 0.2 on the vertical axis for better visibility.

**Figure 3:**
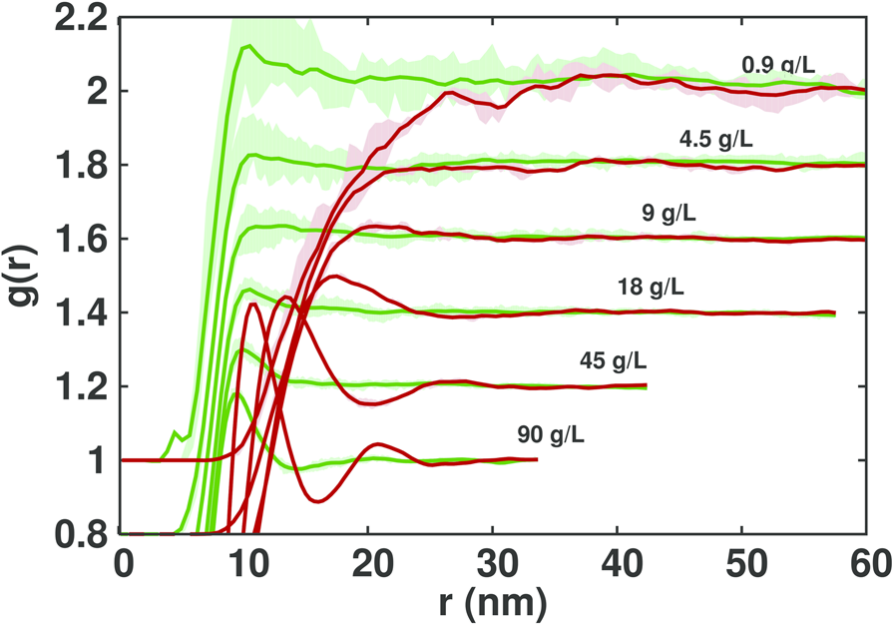
**BSA radial distribution functions.** Computed radial distribution functions at various concentrations (indicated on the plot) of BSA obtained from simulations without (dark green) and with (dark red) the Debye-Hückel approximation. Curves are shifted by 0.2 on the vertical axis for better visibility. Averages and standard deviations of the g(r) are shown by the dark line and light color, respectively.

As shown in Figure 1, the scattering intensities obtained from the simulations with the Debye-Hückel approximation reproduce experimental SAXS intensities better then the intensities calculated from simulations which do not include the Debye-Hückel interaction. In particular, the greatest improvement is seen at low *q* values, i.e. long range interactions are accurately captured. At high concentrations, the Debye-Hückel approximation tends to overestimate the height of the correlation peak seen in the normalized experimental intensities. This phenomenon can be explained considering that simulations have been performed at 5 mM ionic strength, but that at high protein concentrations, the effective ionic strength may be higher due to the presence of highly charged proteins. Indeed, the correlation peak is lower in the simulations without the Debye-Hückel approximation (see also Figure 2 and Figure 3). This suggests that at low ionic strength and high protein concentration, the ionic strength of the simulation should be slightly increased to better reproduce experimentally observed scattering intensities.

The computed static structure factors obtained from the two sets of simulations are compared in Figure 2. Focusing on the low *q* region (*q *< 0.1 *nm*^-1^), for a given concentration, the value of *S*(*q*) is lower when the Debye-Hückel potential is used. The long wavelength limit of *S *(*q*) is proportional to the normalized isothermal osmotic compressibility, vis.:

$$\lim_{q\to 0}S(q)=n_{p}k_{B}T\chi_{T}$$

where *χ*_*T *_is the isothermal osmotic compressibility. (In the canonical ensemble, $\chi_{T}=-V{\left(\frac{\partial V}{\partial \Pi }\right)}_{T}={\left\{n_{p}{\left(\frac{\partial \Pi }{\partial n_{p}}\right)}_{T}\right\}}^{-1}$), *n*_*p*_ is the protein number density, and *k*_*B*_ is the Boltzmann constant [32,41,42]. The decrease of *S *(*q*) at low *q* values can be explained by the decrease of the osmotic compressibility due to the long-range electrostatic repulsion introduced with the Debye-Hückel potential [43].

The first peak in the *S*(*q*) represents the correlation between a pair of proteins. We observe that the simulations which include the Debye-Hückel potential show a shift of the first peak to lower *q* values (at high concentrations) or the appearance of a peak (at low concentrations), indicating the presence of a long-range correlation between the proteins. With increasing concentration, the peak shifts to higher *q* values, suggesting a decrease of the correlation distance. The same effect can be seen better in real space from the radial distribution functions plotted in Figure 3 where it can be seen that the introduction of a long-range repulsion pushes the proteins away from each other. It also leads to a more structured solution, with the appearance of a second peak in the simulations at 90 g/L protein concentration.

##### 

**Figure 4:**
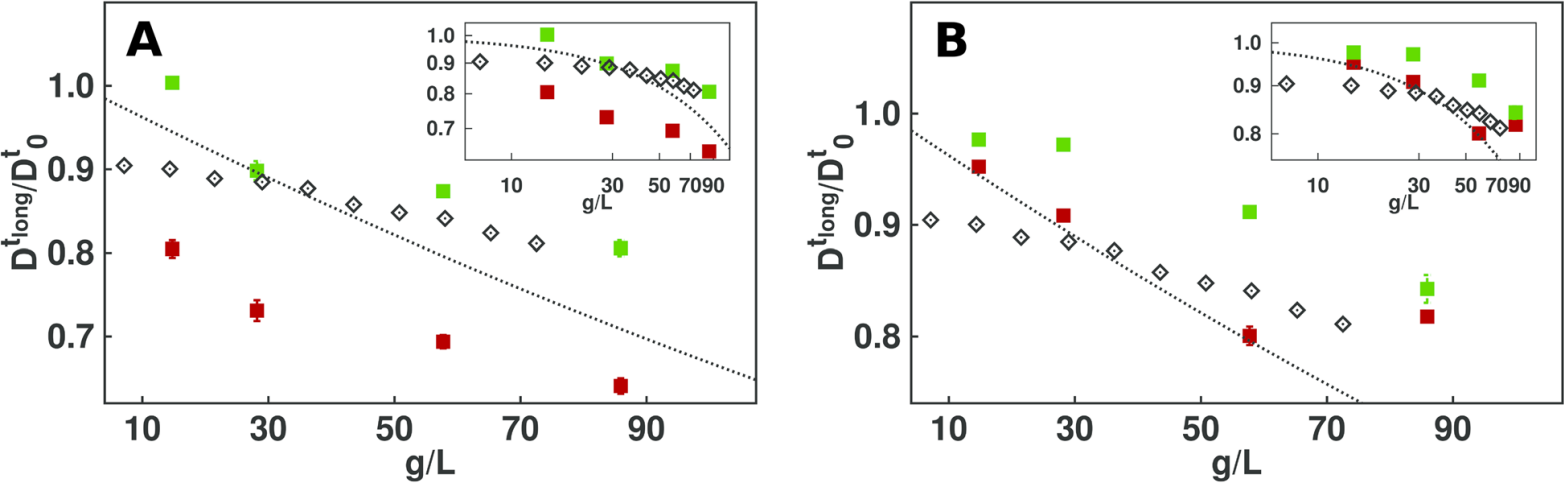
**HEWL translational diffusion coefficients.** Normalized long-time translational self-diffusion coefficients of HEWL at low ionic strength. Simulations were performed at 1 mM **(A)** and 5 mM **(B)** ionic strength. Experimental values from ref. [35] (black diamonds), and computed values from BD simulations with (red squares) and without (green squares) Debye-Hückel potential are shown. The Tokuyama [22] analytical model is shown by the black dotted line. Insets are log-log plots of the same data.

**Long-time self-diffusion coefficients** Besides the effect on protein-protein interactions, the addition of the Debye-Hückel potential also has consequences for the dynamics of the proteins. Simulations of HEWL were performed at low ionic strength (1 and 5 mM) at different lysozyme concentrations and compared to experimental diffusion coefficients obtained from pulsed gradient spin echo NMR for HEWL solutions without added salt at pH 4.9. As shown in Figure 4, the presence of the Debye-Hückel potential systematically lowers the long-time self-diffusion coefficients. This effect can be explained considering that, for a given concentration, simulations which include the Debye-Hückel potential correspond to a larger effective concentration due to the long-range repulsive interaction [43,44]. In general, the magnitude of the effect on the diffusion coefficient due to the Debye-Hückel potential is related to the ionic strength of the solution, the size of the protein, and the protein concentration. For proteins whose size is comparable to the Debye length, *κ*^-1^, as in our case, this effect can be significant. For very large proteins, the Debye length can be much smaller than the size of the protein, and hence, adding the long-range Debye-Hückel interaction may lead only to small effects on the diffusion coefficient.Simulations performed at 1 mM ionic strength underestimate the diffusion coefficients compared to the experimental values (see Figure 4). As described above for the BSA case, the ionic strength of the solution is affected by several factors. Thus, it is possible that the value of 1 mM used in the simulations does not correctly describe the effective ionic strength of the experimental solutions. We therefore also performed simulations at higher ionic strength (5 mM), obtaining better agreement with the experimental data, see Figure 4.

### Methodological considerations

The Debye-Hückel potential has been implemented together with cubic grids for the proteins. The transition from the gridded potential to the Debye-Hückel potential with increasing distance from a solute center occurs at the shortest distance to the grid boundary. Thus, cubic grids permit the most efficient implementation of the Debye-Hückel correction. Their use is usually appropriate for globular proteins, however, it may become an issue when modeling large elongated molecules. For the latter, a large number of grid points on a cubic grid will have very low (negligible) values of the mapped interaction potentials, leading to an unnecessarily high memory requirement.

On the other hand, an advantage of the Debye-Hückel implementation is that it removes the requirement for the electrostatic potential to have very small values at the grid edges; the electrostatic potential is only required to be centrosymmetric. This means that smaller grids can be used with the long-range interactions being captured by the Debye-Hückel with only a small computational cost (see Additional file 2).

Using the Debye-Hückel correction may be an issue for some highly or non-uniformly charged systems as it can lead to force discontinuities at the grid boundaries. A possible solution to this problem, currently not implemented, is to apply an interpolation function between the electrostatic potential grid and the Debye-Hückel potential for computing the forces at the grid boundary.

## Conclusions

We have here described the implementation of a Debye-Hückel correction for the computation of grid-based electrostatic interaction energies and forces for use in atomically detailed many-protein Brownian dynamics simulations. The ability of this many-protein BD method to correctly reproduce small angle scattering data and diffusion coefficients, was previously shown for several proteins [3,12]. Due to computational limitations on the size of the electrostatic interaction grids, the method could not be applied to highly charged systems or low ionic strength conditions without impairing the accuracy of the resulting simulations. The introduction of the simple Debye-Hückel correction described in this paper with its very low associated computational costs allowed us to extend the range applicability of this BD method to highly charged systems at low ionic strength. In particular, comparison of the model with the Debye-Hückel correction to analytical results for spherical solutes, as well as to experimental SAXS intensities for BSA protein solutions, and to long-time self-diffusion coefficients of HEWL protein solutions, showed good agreement. Some other potential applications of the methodology are the simulation of protein crystallization, of protein-surface adsorption, and of heterogeneous crowded protein solutions. Furthermore, the Debye-Hückel correction described here should be of value in implicit solvent molecular dynamics simulations which make use of gridded interaction potentials [13-16].

## Competing interests

The authors declare that they have no competing interests.

## Authors’ contributions

PM and RCW devised and planned the project. PM and MM implemented the method. PM carried out the simulations. PM, MM and RCW analyzed the data. PM and RCW wrote the paper. All authors read and approved the final manuscript.

## Supplementary Material

Additional file 1 **Charged spheres electrostatic potential energy.** Electrostatic potential energy of two uniformly charged spheres for different net charge (e) combinations. Panels A,B: +1/+1, +1/-1; panels C,D: +5/+5, +5/-5; panels E,F: +10/+10, +10/-10. Red and green colors show interactions between charges of the same and opposite sign, respectively. The Debye-Hückel analytical approximation, Equation 4 (continuous line), Brownian dynamics without Debye-Hückel term (crosses) and Brownian dynamics with Debye-Hückel term (circles) are shown. Interactions are computed at 5 mM (left panels) and 300 mM (right panels). The abscissa is the particle center-to-center distance divided by the particle diameter. The inclusion of the Debye-Hückel potential recovers the dependence of the energy on particle separation computed with the analytical model. Click here for file

Additional file 2 **Runtime versus protein concentration for the simulations of BSA.** The Debye-Hückel correction requires very little additional computational effort. Indeed, at low concentrations, the inclusion of the Debye-Hückel correction keeps the like-charged molecules apart and therefore reduces the number of pairs of molecules for which grid-type potentials are used to compute intermolecular forces, thus leading to reduced run-times. Click here for file
