# Correction to: Long range Debye-Hückel correction for computation of grid-based electrostatic forces between biomacromolecules

**DOI:** 10.1186/s13628-020-0026-z

**Published:** 2020-02-11

**Authors:** Paolo Mereghetti, Michael Martinez, Rebecca C. Wade

**Affiliations:** 1 Molecular and Cellular Modeling Group, Heidelberg Institute for Theoretical Studies (HITS), Schloß-Wolfsbrunnenweg 35, 69118 Heidelberg, Germany; 2 Center for Nanotechnology Innovation@NEST, Italian Institute of Technology, Piazza San Silvestro 12, Pisa, Italy; 3 Center for Molecular Biology (ZMBH), University of Heidelberg, Im Neuenheimer Feld 282, 69120 Heidelberg, Germany

**Correction to:** ***BMC Biophys*** **7, 4 (2014)**

**doi:10.1186/2046-1682-7-4**

The original publication of this article [1] contained an error. This error is due to the misassignment of one value (the molecular weight of hen egg white lysozyme (HEWL)).

In the section “**BD simulations of protein solutions**”, the concentrations of HEWL given for the simulations are incorrect.

The incorrect and correct text is as followed.

1. **Incorrect**
  - Simulations of HEWL were performed at 14, 28, 57 and 85 g/L for comparison with experimental long-time translational self-diffusion coefficients [35]
2. **Correct**
  - Simulations of HEWL were performed at 3.1, 6.1, 12.3 and 18.4 g/L for comparison with experimental long-time translational self-diffusion coefficients [35]

Consequently, in Figure 4, the computed normalized HEWL translational diffusion coefficients (green and red squares) are shown at the wrong HEWL concentrations. They should be shown at HEWL concentrations of 3.1, 6.1, 12.3, and 18.4 g/L instead of 14.3, 28.6, 57.2 and 85.8 g/L, respectively.

The methodology introduced in this paper and the overall conclusions are not affected by this correction.
