# Supplementary figures and images for: Long range Debye-Hückel correction for computation of grid-based electrostatic forces between biomacromolecules
Source: BMC Biophys. 2014 Jun 17;7:4. doi: 10.1186/2046-1682-7-4 (PMC4082500; doi:10.1186/2046-1682-7-4)

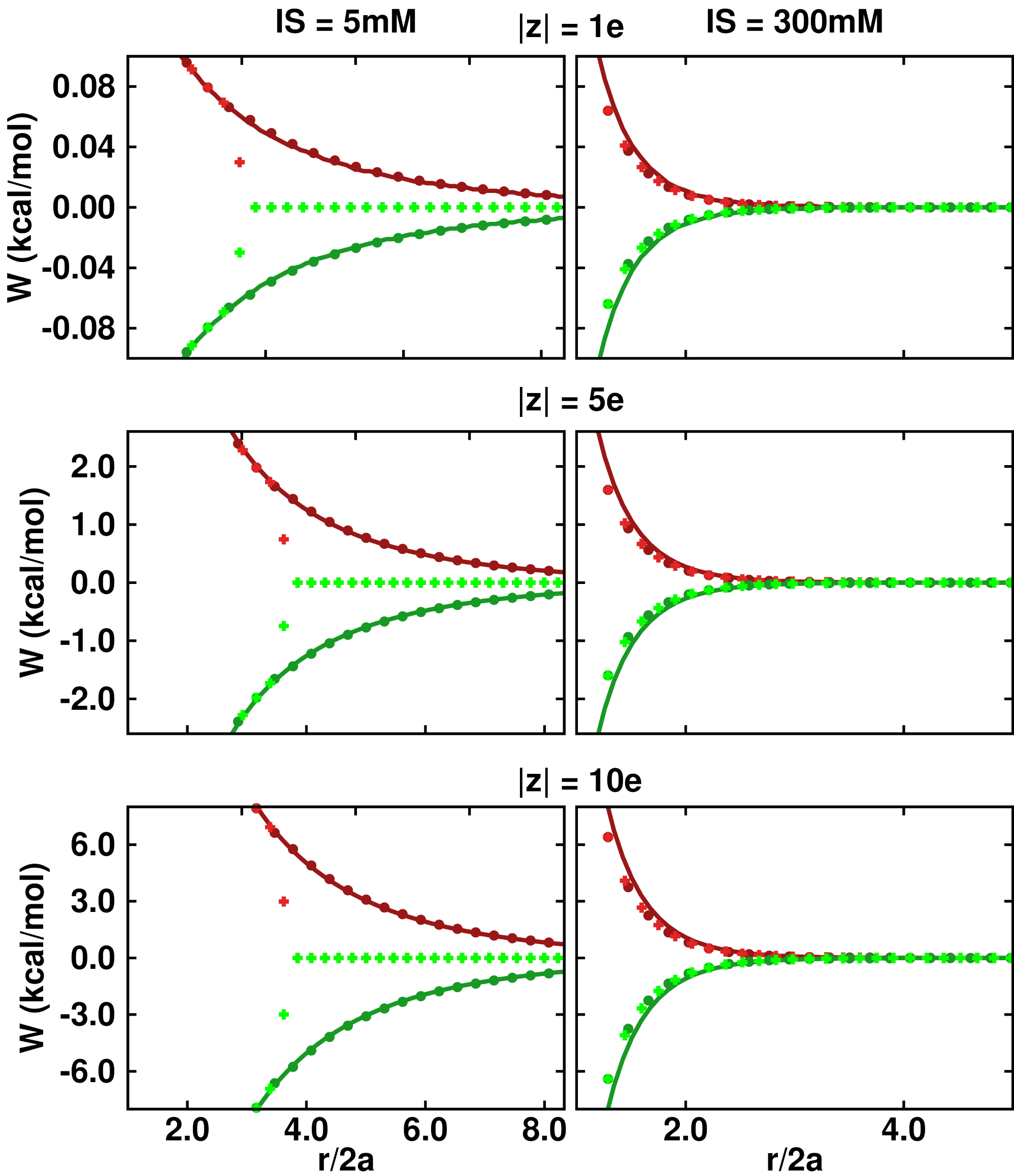

Supplement: Additional file 1 — Charged spheres electrostatic potential energy. Electrostatic potential energy of two uniformly charged spheres for different net charge (e) combinations. Panels A,B: +1/+1, +1/-1; panels C,D: +5/+5, +5/-5; panels E,F: +10/+10, +10/-10. Red and green colors show interactions between charges of the same and opposite sign, respectively. The Debye-Hückel analytical approximation, Equation 4 (continuous line), Brownian dynamics without Debye-Hückel term (crosses) and Brownian dynamics with Debye-Hückel term (circles) are shown. Interactions are computed at 5 mM (left panels) and 300 mM (right panels). The abscissa is the particle center-to-center distance divided by the particle diameter. The inclusion of the Debye-Hückel potential recovers the dependence of the energy on particle separation computed with the analytical model. [file 2046-1682-7-4-S1.pdf]

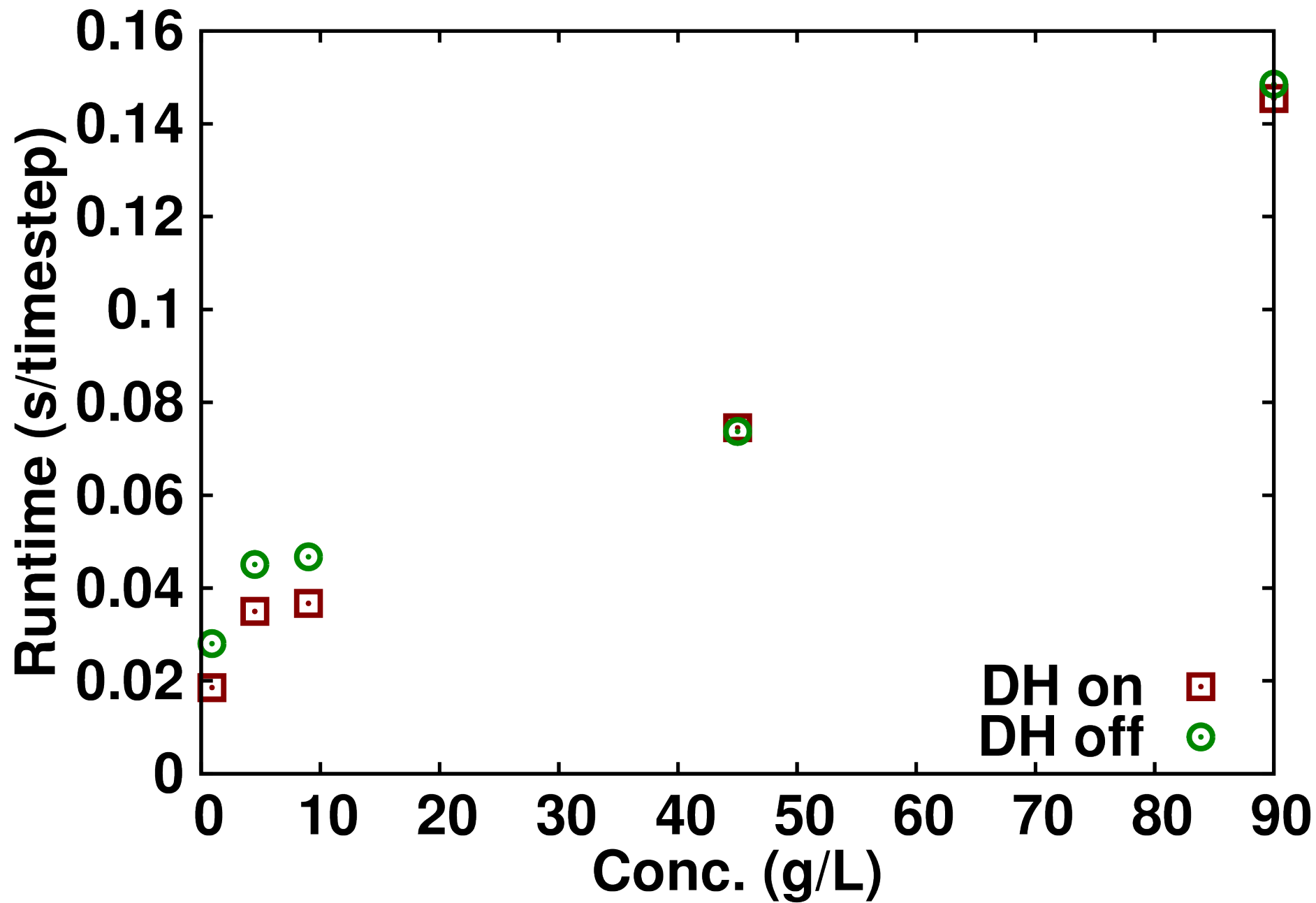

Supplement: Additional file 2 — Runtime versus protein concentration for the simulations of BSA. The Debye-Hückel correction requires very little additional computational effort. Indeed, at low concentrations, the inclusion of the Debye-Hückel correction keeps the like-charged molecules apart and therefore reduces the number of pairs of molecules for which grid-type potentials are used to compute intermolecular forces, thus leading to reduced run-times. [file 2046-1682-7-4-S2.pdf]
